# Intraoperative quality assessment of tissue perfusion with indocyanine green (ICG) in a porcine model of mesenteric ischemia

**DOI:** 10.1371/journal.pone.0254144

**Published:** 2021-07-20

**Authors:** Anna Duprée, Henrik Rieß, Philipp H. von Kroge, Jakob R. Izbicki, Eike S. Debus, Oliver Mann, Hans O. Pinnschmidt, Detlef Russ, Christian Detter, Sabine H. Wipper

**Affiliations:** 1 Department General, Visceral and Thoracic Surgery, University Medical Center Hamburg-Eppendorf, Hamburg, Germany; 2 Department of Vascular Medicine, University Heart Center, University Medical Center Hamburg-Eppendorf, Hamburg, Germany; 3 Department of Medical Biometry and Epidemiology, University Hospital Eppendorf, Hamburg, Germany; 4 Department for the Development of Applications, Institute for Laser Technology, University Ulm, Ulm, Germany; 5 Centre of Cardiology and Cardiovascular Surgery, University Hospital Eppendorf, Hamburg, Germany; Dipartimento di Scienze Mediche e Chirugiche (DIMEC), Orsola Hospital, ITALY

## Abstract

**Background:**

Mesenteric ischemia is a severe and potentially lethal event. Assessment of intestine perfusion is eminently depending on the skills, and the experience of the surgeon. Thus, the therapy is biased by the right evaluation. Aim of this study is to determine the applicability, and the usefulness of fluorescent-imaging (FI) with indocyanine green (ICG) in a porcine model of mesenteric ischemia. Second end-point is the verification of a visual and quantitative assessment tool of the intestinal perfusion.

**Methods:**

In 18 pigs (54,2 ±2,9kg) an occlusion of a side-branch of the mesenteric artery was performed for 3 (group I, n = 7), 6 (group II, n = 7), and 10 hours (group III, n = 4). After reperfusion a 60 minutes observation period was carried out. 3 regions of interest were defined: ischemic bowel (D1), transitional zone (D2), and non-ischemic bowel (D3). ICG-FI was performed during baseline (T0), occlusion (T1), reperfusion (T2) and after an observation period of 60 minutes (T4).

**Results:**

All experiments could be finished successfully. ICG-FI was assessed using assessment of background-subtracted peak fluorescence intensity (BSFI), slope of fluorescence intensity (SFI), and a baseline adjusted ratio of both parameters. ICG-FI confirmed loss of perfusion in D1, decreased perfusion in D2, and increased perfusion in D3. After reperfusion ICG-FI increased in group 2 due to a severe tissue damage resulting in a capillary leakage. In group I ICG-FI was equal to baseline values indicating the totally reversible loss of perfusion.

**Conclusion:**

Using ICG-FI to estimate intestine perfusion after different durations of ischemia is viable using a porcine model of mesenteric ischemia. Even small differences in perfusion can be reliably determined by ICG-FI. Thus, ICG-FI is an encouraging method to evaluate intestine perfusion intraoperatively.

## Introduction

Mesenteric ischemia is still a lethal disease. Fast diagnosis and therapy results in an improvement of prognosis. The high mortality of this disease, ranging between 60–80%, has not changed significantly in the last few years [1–4], despite improved and complex treatment strategies [5, 6].

Direct surgery is commonly recommended as the treatment of choice. Previous diagnostics often only delay proper treatment [6]. During the first operation, a resection should be limited to the minimum to avoid the resection of intestine with the potential to regenerate. Whether there is a potential for tissue regeneration, and what criteria can be used to assess it remains vague. The intraoperative decision making about potentially reversible ischemic areas is subjective, and highly depends on the experience of each surgeon [7]. Despite regular appearance of intact serosa, mucosal ischemia can still be present, leading to undervalue extent of tissue destruction. Thus, scheduled second look operations should be applied up to 48 hours later to reevaluate and resect irreversible necrotic parts [7, 8].

Still there is no standardized technique to evaluate tissue perfusion status during mesenteric ischemia. Indocyanine green fluorescent imaging (ICG-FI) is a technology for visual and quantitative evaluation of tissue perfusion in various fields [9–15]. Currently, ICG-FI is of increasing interest for intraoperative quality control in visceral surgery. Up to now mainly visual interpretation ICG-FI was performed. Only few studies tried to establish quantitative assessment [16, 17]. Detter et al. previously described two quantitative assessment tools for assessment of myocardial perfusion in graded coronary artery stenosis [18], and coronary artery bypass stenosis [19]. In a previous study we validated these assessment tools in a one-vessel-model [20]. Aim of this study was to determine the applicability, and the usefulness of fluorescent-imaging (FI) with indocyanine green (ICG) in a porcine model of mesenteric ischemia. Second end-point is the verification of a visual and quantitative assessment tool of the intestinal perfusion.

## Methods

This study was carried out in stringent conformity with the recommendations in the Guide for the Care and Use of Laboratory Animals of the National Institutes of Health. The protocol was authorized by the Committee on the Ethics of Animal Experiments of the Authority for Health and Consumer Protection Hamburg (Behörde für Gesundheit und Verbraucherschutz, Hamburg, Protocol Number: 113/14). The experiments were performed under deep sedation. All attempts were made to minimize suffering.

The experiments were performed in 18 pigs of either sex weighing 54.2 +/- 2.9 kg at the Institute for Surgical Research, University Center Hamburg-Eppendorf (Hamburg, Germany).

Premedication and anesthesia was performed as previously described [21]. Briefly, after intramuscular premedication with azaperone (4 mg/kg), midazolam (0.3 mg/kg), ketamine (5 mg/kg), and atropine sulfate (0.15 mg/kg), intravenous anesthesia was induced by propofol (0,06 mg/kg) and maintained by continuous infusion of fentanyl (0.01 mg/kg/h), midazolam (0.1 mg/kg/h), ketamine (0,1mg/kg/h), and propofol (3 mg/kg/h). The animals were endotracheally intubated and pressure-controlled ventilated at 15 cm H_2_O with a positive end-expiratory pressure of 7 cm H_2_O at 16 breaths per minute using 30% oxygen. A central venous catheter was placed into the right jugular vein for volume substitution, and central venous pressure monitoring.

A 4F arterial catheter was inserted into the right femoral artery for continuous hemodynamic monitoring documented by the PiCCO device (Pulsion Medical Systems, Munich, Germany).

### Surgical procedures

**Fig 1:**
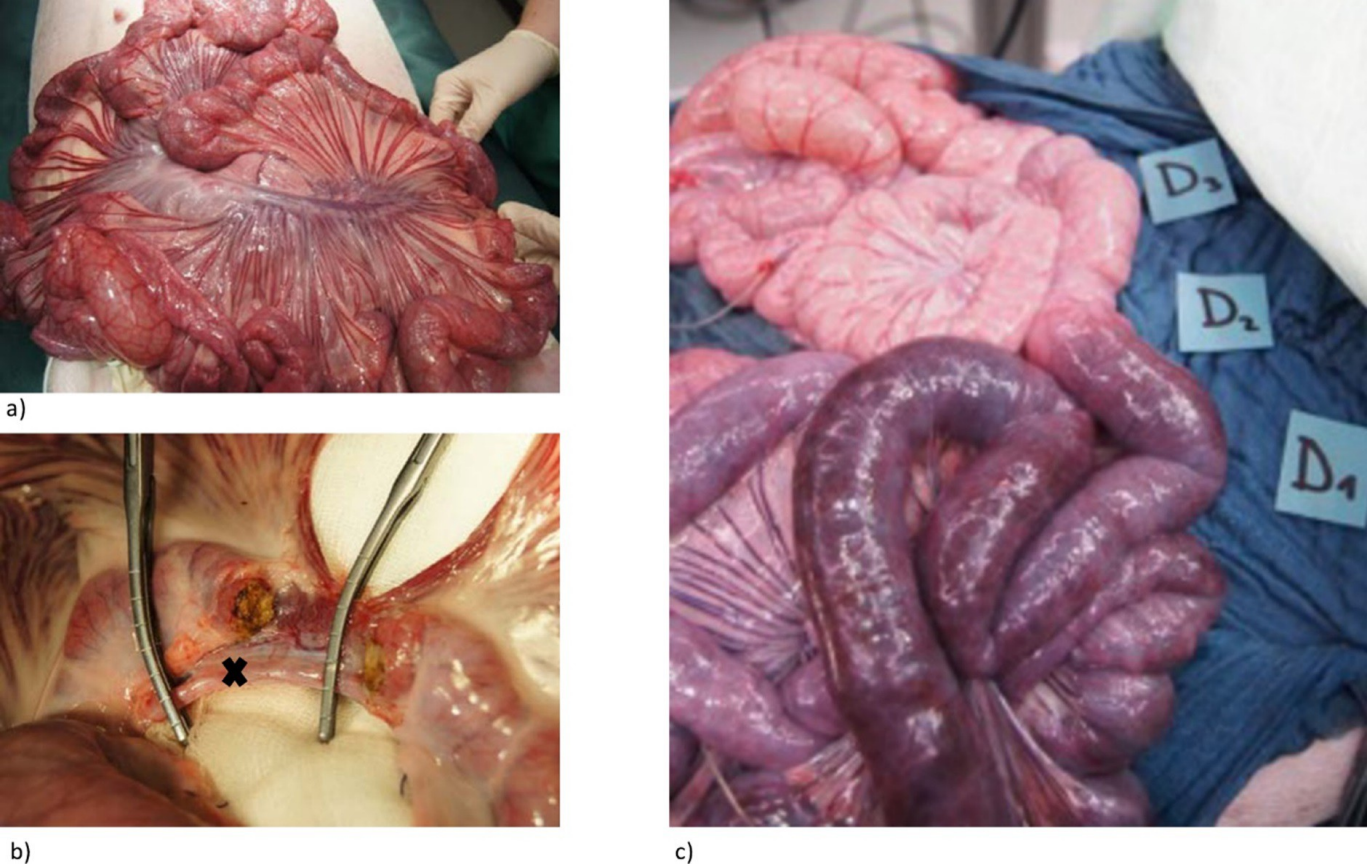
Model of mesenteric ischemia. A standardized model for mesenteric ischemia was previously established in an unpublished pilot study. Briefly, a huge branch of the superior mesenteric artery (SMA) was dissected in the meso-ileum. Due to huge venous and arterial collateralization in pigs (a), a complete dissection of the depending mesentery was performed, including the mesenteric vein and its venous weave (b). Afterwards the dissected branch of superior mesenteric artery (✖) is clamped to induce ischemia. For detailed evaluation, 3 ROIs were defined in ischemic (D1), transitional (D2), and control areas (D3), corresponding to the degree of ischemia (c).

Midline laparotomy was performed. A standardized model for mesenteric ischemia was previously established in an unpublished pilot study. Briefly, a huge branch of the superior mesenteric artery (SMA) was dissected in the meso-ileum next to the ileocecal junction. Due to huge venous and arterial collateralization, a complete dissection of the depending mesentery was performed, including the mesenteric vein and its venous weave (Fig 1). For steady measurement of intestine blood flow, a Doppler flow probe (CardioMed Flowmeter, Medi-Stim AS, Oslo, Norway) was placed around the dissected branch of the SMA. After baseline assessment, selective clamping of the dissected arterial and venous branches was performed (Fig 1).

### Experimental protocol

**Fig 2:**
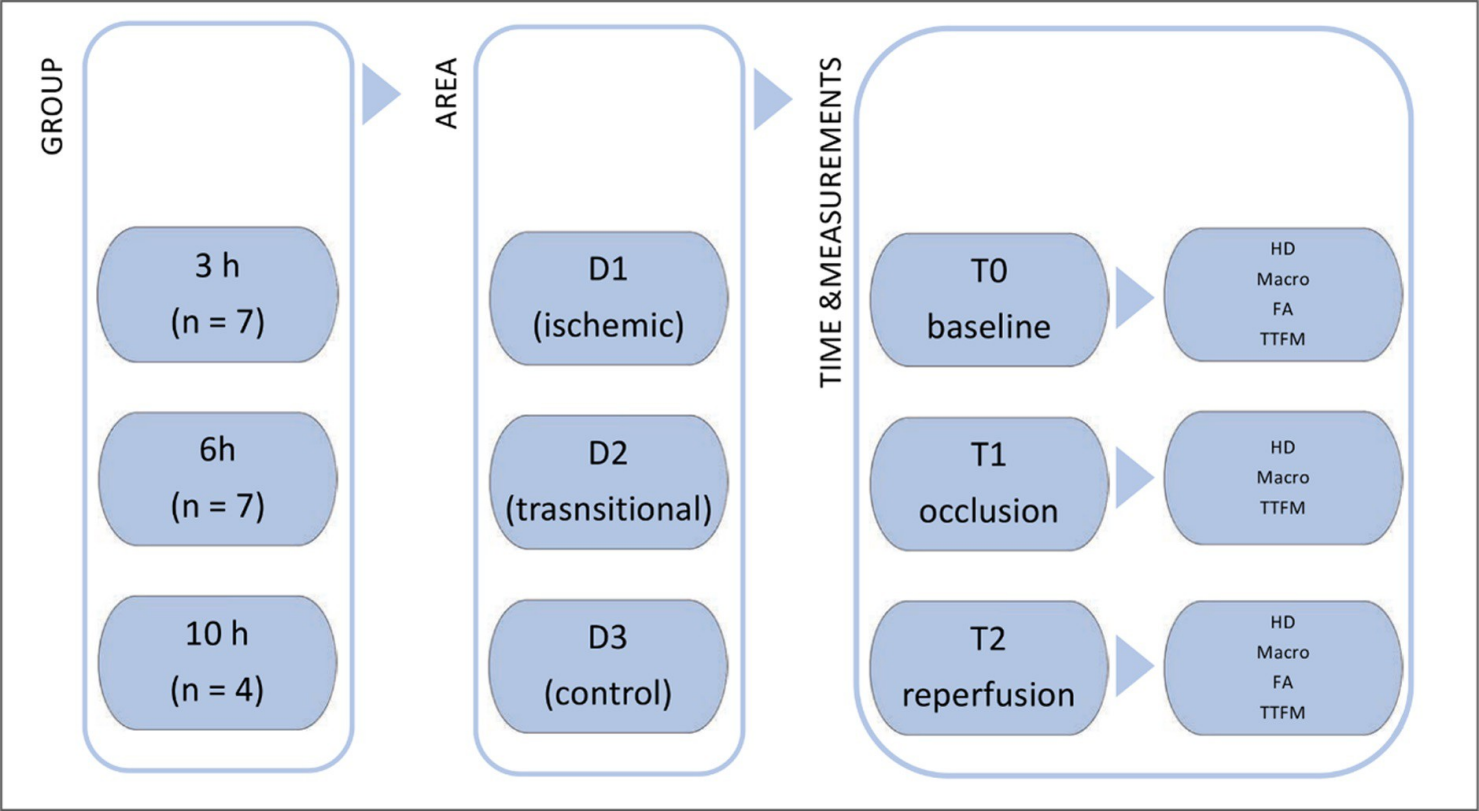
Experimental setting. This figure shows the simplified experimental set up. In each group different ischemic durations were studied. Number of animals are shown in the brackets. In each animal different areas were defined in order to their perfusion status. In each area, the measurements were carried out at the three time points T0-T1. h: hours of ischemia;n: number of individuals; D1: ischemic area; D2: transsitional area; D3: control area; T0: baseline;T1:occlusion;T2:reperfusion; HD:hemodynamics;Macro: macroscopic score; FI: Fluorescent Imaging; TTFM: Transit Time Flow Measurement in Superior Mesenteric Artery.

Pigs were randomized into 3 groups with total ischemia duration of 3 (group I, n = 7), 6 (group II, n = 7), and 10 hours (group III, n = 4). 3 regions of interest (ROIs) were defined in ischemic (D1), transitional (D2), and control areas (D3) (Fig 2).

Hemodynamic parameters, and metabolic parameters were documented hourly. Semi-objective assessment of each area was performed hourly by two experienced visceral surgeons using a macroscopic score including color, peristalsis, edema formation, and mesenteric hemorrhage. The degree of ischemia was categorized into none/mild (<6 points), moderate (6–9 points), and severe (>9 points).

ICG-FI was performed with the FCI system (LLS GmbH) developed by the institution of laser technology of the university Ulm [22]. ICG-FI and TTFM were performed during baseline before ischemia induction (T0), during occlusion (T1), and after reperfusion (T2). After ischemia, reperfusion was induced by declamping. Parameters were collected at peak of hyperperfusion assessed by Transit Time Flow Measurement (TTFM), and thereafter every 15 minutes up to 60 minutes after reperfusion. Pigs were sacrificed by intravenous injection of T61 in deep anesthesia.

### Fluorescent imaging

**Fig 3:**
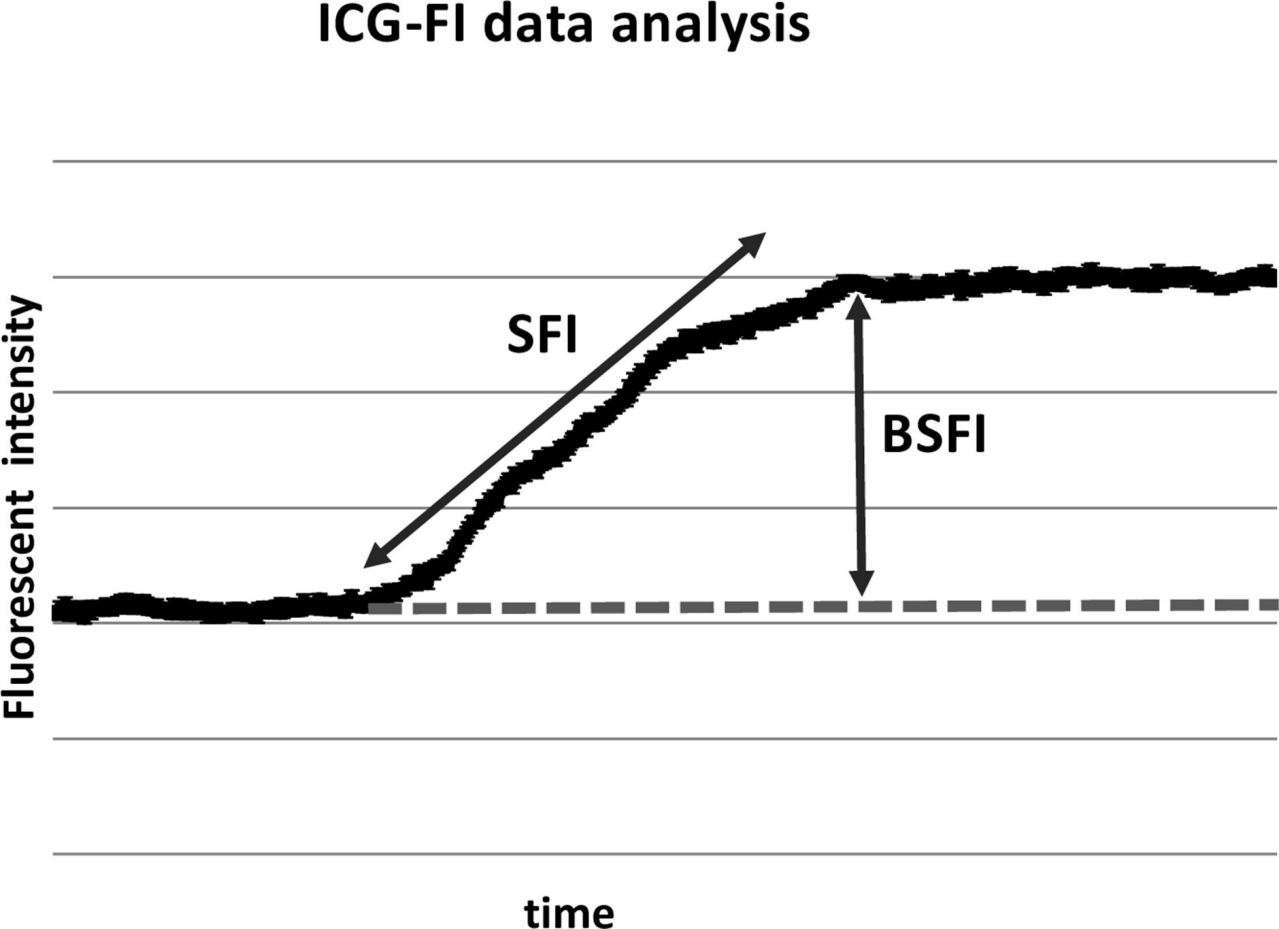
Technique of quantification. ICG-FI data is analyzed in the corresponding fluorescent intensity curve of each ROI. ICG-FI: Indocyanine green fluorescent imaging; ROI: region of interest; BSFI: Backround substracted fluorescent intensity; SFI: Slope of fluorescent intensity.

The technique of ICG-FI and data analysis was previously described in detail by Detter et al 2007 [18]. Briefly, the corresponding fluorescent intensity curves in each ROI (D1-D3) were analyzed. Mean value and standard deviation (SD) were calculated for each ROI in a sequence of 60 seconds after the injection of the ICG. To distinguish bowel perfusion, 2 different parameters derived from the time-dependent fluorescent curve were defined (Fig 3):

#### Background-Subtracted Peak Fluorescence Intensity (BSFI)

To calculate BSFI from the time-dependent fluorescence intensity curves, the initial intensity value before the injection of ICG was subtracted from the peak fluorescence intensity during the first passage of the dye through the bowel segment [18].

#### Slope of Fluorescence Intensity (SFI)

This parameter is represented by the maximal slope during the increase of the time-dependent fluorescence intensity induced by the first wave of the dye, which passes the capillaries of the bowel tissue.

Each ICG-FI sequence was recorded online for 120 seconds with real-time digitizing. BSFI and SFI data were analyzed offline directly after the images were recorded with a customized software package (LLS GmbH).

### Histopathology

Representative specimen of the bowel in the ischemic, transitional, and control area were removed, and thereafter stored in 3.5% buffered formalin. All specimens were routinely processed and embedded in paraffin. 5-μm slices were stained with hematoxylin and eosin. And examined blinded to the treatment using an established scoring system [23].

### Statistics

Data of continuous variables were presented as means and standard deviations (SD) across animals. Data distribution was visually assessed by histograms and box plots. The variables BSFI, SFI, SFI-ratio and BSFI-ratio were ln-transformed prior to further analyses to minimize skewness and heteroscedasticity. The means of these ln-transformed variables, along with their 95% confidence intervals, were graphically presented by group, location and time. Category counts of variable “macro score” were also graphically presented by group, location and time. Data were then subjected to mixed model analyses to take repeated measurements within animals into account. For the dependent variables BSFI and SFI, we fitted initial models containing fixed effects of group, areal, time, and areal-by-time. Random intercepts for raters and animals were fitted and repeated measures over time within areal-by-animal-by-rater were accounted for. For the dependent variables tpO_2_, fixed effects of group, areal, time and areal-by-time were tested, accounting for repeated measures over time within areal-by-animal. For the dependent variable “flow” and the hemodynamic dependent variables CO, CI, HR, MAP, SVR, GEDV and GVD a fixed effect for time was fitted and repeated measures over time within animal were accounted for. Non-significant fixed effect terms were excluded from the models using a hierarchical stepwise-backwards approach. For repeated measures, an autoregressive covariance structure was employed while a variance components covariance structure for estimating random intercepts. Time was treated as a categorical variable. A value of p<0.05 was considered statistically significant. Statistical analysis was performed with the SPSS statistical software package 23.0 (SPSS Inc, Chicago, Ill).

## Results

Experiments have been performed successfully in all 18 pigs. None of the pigs had to be excluded.

Hemodynamics were stable between the groups throughout the whole experiment without significant differences between the groups. However, there were slightly higher cardiac output values at baseline in group III, while systemic vascular resistance was highest in group I (n.s.).

### Transit-Time Flow Measurement (TTFM)

TTFM flow was comparable in all 3 groups during T0 (69.7± 16.5 ml/min), decreased to 0±0ml/min during complete occlusion, and increased immediately after reperfusion to highest values (group I: 60.7 ± 48.4 ml/min, group II: 97.1 ± 31.0 ml/min, group III: 142.5 ± 60.0 ml/min, p<0.05). Compensatory hyperemia was highest in group 3 and lowest in group 1. Values remained stable over 60 minute observational period.

### Visual assessment

#### Macroscopic score

Macroscopic evaluation showed a worsening of bowel viability in all groups during ischemia. During occlusion, bowel viability in the ischemic area D1 was 11.4 ±1.8 after 3 h in group I, 11.67±1.03 after 6 h in group II, and 12.25±0.96 after 10 h in group III, while maintaining baseline values (4.0±0) in the non-ischemic area D3 in all groups (p<0.05). 60 minutes after reperfusion values in group I decreased to 7.0 ±1.41, in group II to 9.33±1.86, and remained at 12.50±1.25 in group III (p<0.05). Values of transitional zone D2 were significantly worse than in the non-ischemic area D3 (p<0.05) during occlusion. However, macroscopic Score in D2 was significantly better than the ischemic area D1 at the same time (9.71±0.76 in group I; 10.33±1.03 group II; 12.0±1.16 in group III; p<0.05), as well as during 60 minutes after reperfusion (6.43±1.27 in group I; 9.5±2.95 in group II; 12.0±1.83 in group III; p<0.05). While macroscopic aspect of the bowel tissue improved again after reperfusion in the 3 h and 6 h group, irreversible severe damage was evaluated after 10 h of ischemia (p<0.05).

#### Microscopic findings

**Fig 4:**
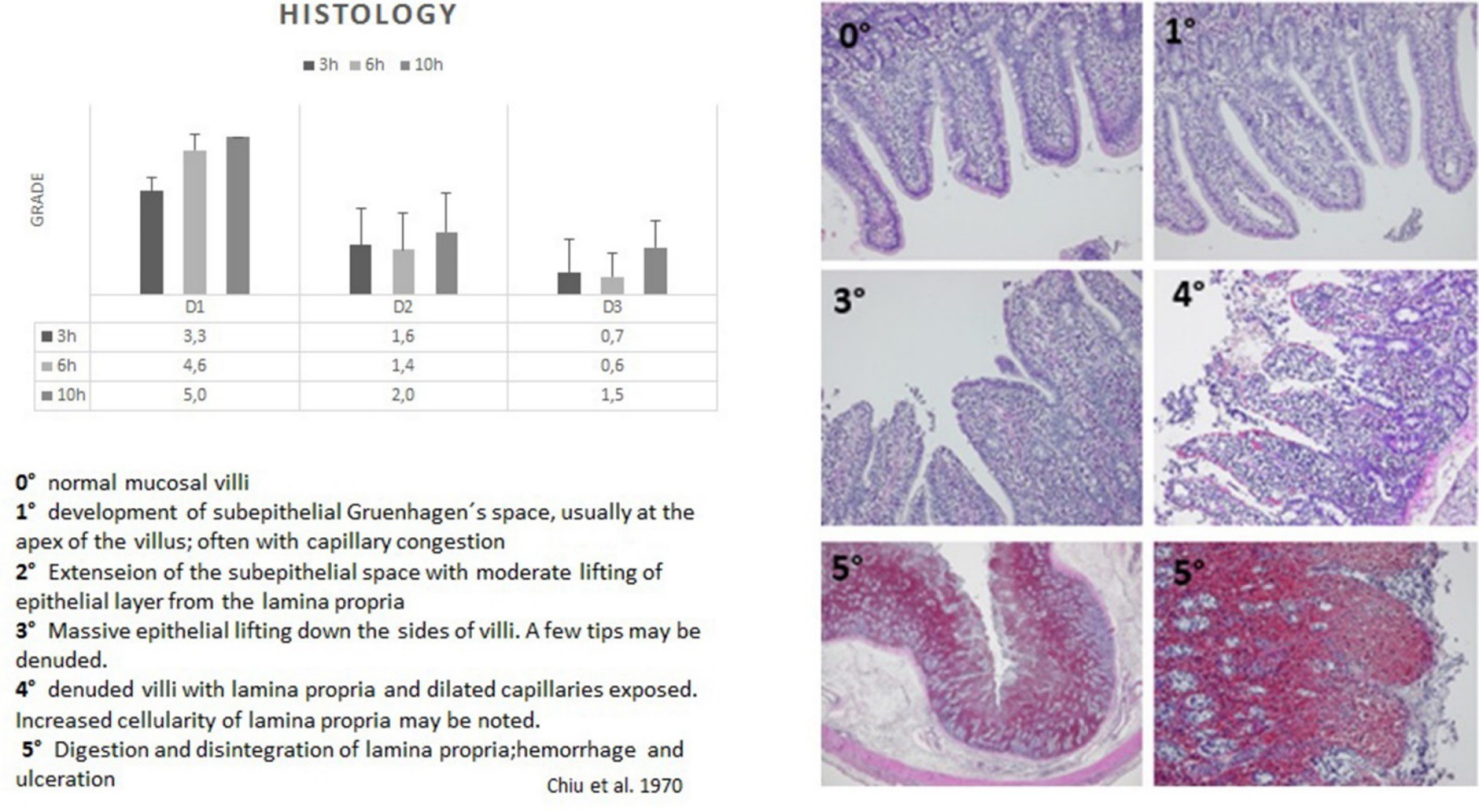
Microscopic findings. Histopathologic examination was performed using the score described previously by Chiu 23,24. Post ischemic bowel showed after 3 h massive epithelial lifting down the sides of villi with denudation of a few tips (grade 3). 6h after ischemia, tissue showed denudation of villi including lamina propria and exposure of dilated capillaries (grade 4), thus indicating principally reversible damages. 10 h of ischemia resulted in digestion and disintegration of lamina propria with hemorrhage and ulceration (grade 5) representing irreversible damage, thus, significantly worse than after 3 h of ischemia (p<0.05). Transitional zone D2, as well as non-ischemic zone D3 showed mild histopathologic signs of epithelial damage in all groups. The histopathological differences between the ROIs reaches significance (p<0.05). (D1:ischemic;D2:transitional zone;D3:Control).

Histopathologic examination was performed using the score described previously by Haglund [24] and Chiu [23]. Post ischemic bowel showed after 3 h massive epithelial lifting down the sides of villi with denudation of a few tips (grade 3). 6h after ischemia, tissue showed denudation of villi including lamina propria and exposure of dilated capillaries (grade 4), thus indicating principally reversible damages. 10 h of ischemia resulted in digestion and disintegration of lamina propria with hemorrhage and ulceration (grade 5) representing irreversible damage, thus, significantly worse than after 3 h of ischemia (p<0.05). Transitional zone D2, as well as non-ischemic zone D3 showed mild histopathologic signs of epithelial damage in all groups (Fig 4). The histopathological differences between the ROIs reaches significance (p<0.05).

#### ICG-FI

Visual assessment of the ICG-FI revealed homogeneous perfusion of the corresponding bowel in all areas at baseline. Occlusion of the dissected SMA branch resulted in a total perfusion defect with no visible fluorescence signal in the corresponding bowel (D1). Visual assessment and evaluation of bowel viability after reperfusion was difficult in all areas, due to variable degree of residual fluorescent signals, depending on the period of ischemia.

### Quantitative assessment

The impairment of perfusion was quantified by BSFI and SFI obtained with ICG-FI.

#### Slope of Intensity (SFI)

*Baseline (T0)*. Baseline values measured by SFI showed incongruent results in between the groups with significantly higher values in group III (group I: 1.27±0.50 group II: 1.93±0.93; group III: 2.60 ± 2.41; p<0.05). The ROIs at baseline showed in all groups significantly higher ICG-FI in D3 (control), and lowest in D2 (transitional) (D1: 1.80±1.01; D2: 1.31±0.69; D3: 1.88±1.66; p<0.05).

*Occlusion (T1)*. Under occlusion ICG-FI decreased significantly in D1 (ischemic) (0.15±0.11; p<0.05), and also in D2 (transitional) (0.62±0.44; p<0.05), while D3 (control) showed similar, but slightly elevated ICG-FI values compared to baseline (3.45±1.74; p = n.s.).

**Fig 5:**
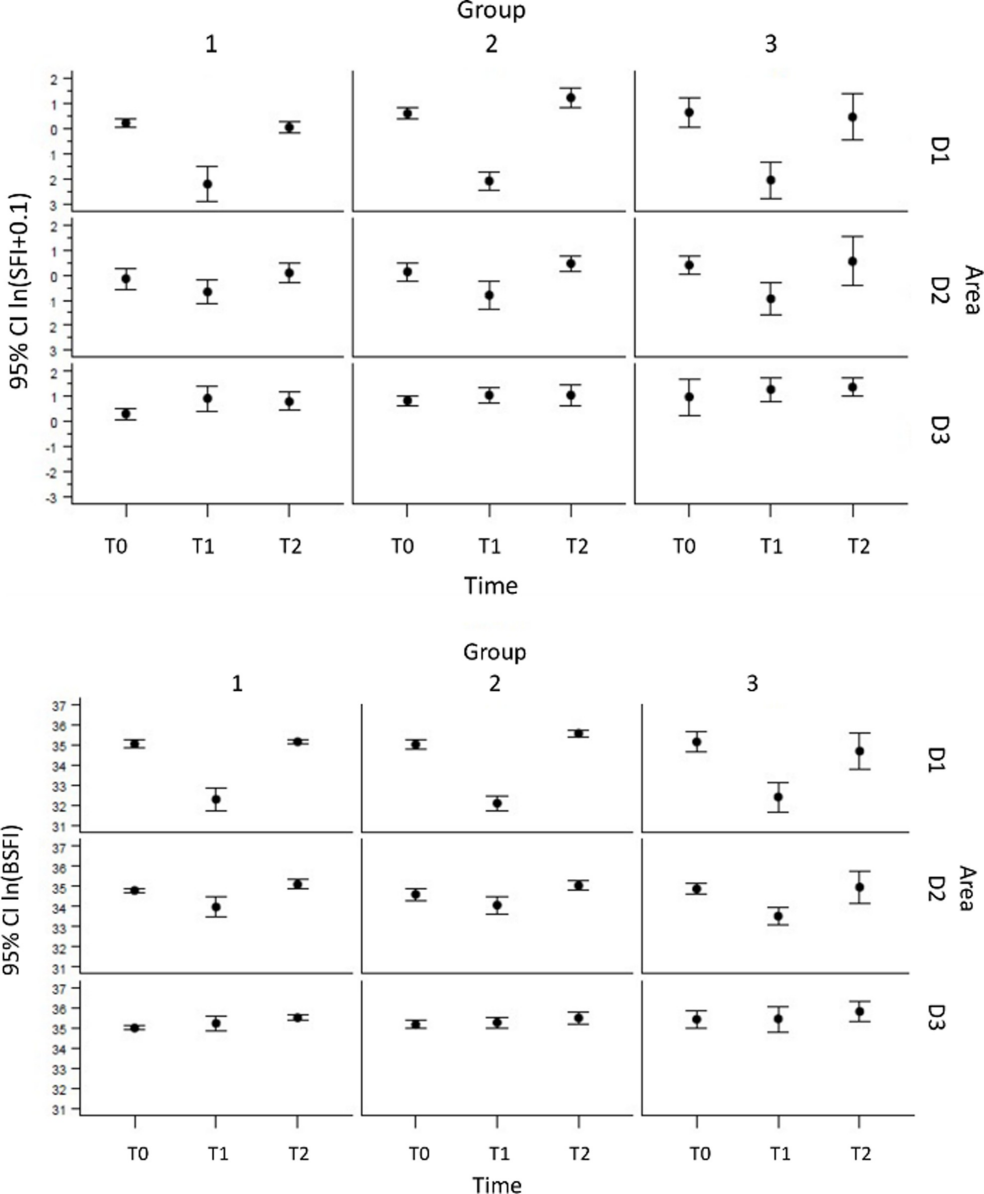
**a.** Assessment of bowel perfusion using ICG- Fluorescent imaging (ICG-FI) using Slope of Fluorescence Intensity (SFI) analysis. This parameter is represented by the maximal slope during the increase of the time-dependent fluorescence intensity induced by the first wave of the dye, which passes the capillaries of the bowel tissue. SFI showed significantly reduced perfusion in D1 (ischemic) and D2 (transitional) under Occlusion (T1) (p<0.05) in all groups, while D3 (control) showed similar results to baseline values. After reperfusion D1 (ischemic) showed enhanced ICG-FI in all groups compared with occlusion (p<0.05), while SFI was lowest after 3 hours of ischemia (group I), and highest after 6 hours of ischemia (group II). ICG-FI in D3 remained high also after reperfusion. P-values for fixed effects were significant for area (pa), and time dependent area (pa*t), but not for time (pt). The graph shows means and 95% confidence intervals of ln-transformed data by area and time. **b.** Assessment of bowel perfusion using ICG- Fluorescent imaging (ICG-FI) using Baseline substracted Fluorescece Intensity (BSFI) analysis. To calculate BSFI from the time-dependent fluorescence intensity curves, the initial intensity value before the injection of ICG was subtracted from the peak fluorescence intensity during the first passage of the dye through bowel tissue. Under occlusion ICG-FI in D1 (ischemic) and D2 (transitional) decreased significantly (p<0.05), while ICG-FI in D3 was significantly elevated compared to D1 and D2, and similar to baseline (p<0.05). ICG-FI in D1 (ischemic) after reperfusion showed significantly enhancement compared to occlusion (p<0.05). Group III showed significantly higher ICG-FI compared to group I and group II after reperfusion (p<0.05). Group I showed similar ICG-FI levels like group III using BSFI analysis. Results were comparable to baseline values, and improved compared to occlusion. P-values for fixed effects were significant for area (pa), time (pt), and time dependent area (pa*t). The graph shows means and 95% confidence intervals of ln-transformed data by area and time.

*Reperfusion (T2)*. After reperfusion D1 (ischemic) showed enhanced ICG-FI in all groups compared with occlusion (group I: 1.16±0.58; group II: 4.08±2.06; group III: 2.58±2.59; p<0.05), while SFI was lowest after 3 hours of ischemia (group I), and highest after 6 hours of ischemia (group II). ICG-FI in D2 (transitional) was significantly impaired compared to baseline (p<0.05), but significantly improved compared to occlusion (group I: 1.39±1.02; group II: 1.78±0.73; group III: 2.63±1.92; p<0.05). ICG-FI in D3 remained high also after reperfusion (group I: 2.61±1.41; group II: 3.69±2.72; group III: 4.30±1.98; p = n.s.) (Fig 5A).

#### Background-Subtracted Fluorescence Intensity (BSFI)

*Baseline (T0)*. BSFI at baseline showed similar results in group I and II (p = n.s.), while ICG-FI in group III was significantly higher (group I: 15.82±4.79; group II: 16.54±7.49; group III: 21.29±12.18; p<0.05).

*Occlusion (T1)*. Under occlusion, BSFI confirmed SFI data. ICG-FI in D1 (ischemic) and D2 (transitional) decreased significantly (p<0.05), while ICG-FI in D3 was significantly elevated compared to D1 and D2, and similar to baseline (D1: 2.04±4.02; D2: 6.42±4.01;D3: 24.72±12.03; p<0.05).

*Reperfusion (T2)*. ICG-FI in D1 (ischemic) after reperfusion showed significantly enhancement compared to occlusion (group I: 19.14±2.80; group II: 29.45±7.76; group III: 19.16±18.94; p<0.05). Similar to SFI results, group III showed significantly higher ICG-FI compared to group I and group II after reperfusion (p<0.05). In contrast to SFI findings, group I showed similar ICG-FI levels like group III using BSFI analysis. BSFI results in D2 (transitional) were comparable in all groups with highest levels in group III (group I: 19.02±7.58; group II: 17.79±6.1; group III: 20.50±12.54; n.s.). Results were comparable to baseline values, and improved compared to occlusion. ICG-FI in D3 (control) remained high with highest values in group III (group I: 27.20±6.81; group II: 29.00±14.33; group III: 40.74±19.30;p<0.05) (Fig 5B).

#### BSFI- and SFI-ratio

To avoid the impact of differences in hemodynamics in between the groups, a ratio to baseline values for each group at occlusion and reperfusion was chosen. Thus, values of 1 are equal to baseline, values > 1 indicate higher ICG-FI, values <1 of lower ICG-FI.

*Occlusion (T1)*. Under occlusion SFI ratio reflected perfusion deficit in D1 in the different groups (group I: 0.11±0.09; group II: 0.09±0.06; group III: 0.08±0.06; p<0.05). D2 showed impaired perfusion in all groups (group I: 0.67±0.31; group II: 0.65±0.77; group III: 0.31±0.18;p<0.05), while D3 showed hyper intensity (group I: 2.47±1.37; group II: 1.55±1.01; group III: 1.88±1.42; p<0.05)).

*Reperfusion (T2)*. After reperfusion perfusion areal D 1 showed nearly equal intensity to baseline in group I (1.04±0.86; p<0.05), while group 2 showed more than doubled values (2.37±1.41; p<0.05). Interestingly Group 3 had similar results to group I with moderate hyper intensity (1.31±1.13;n.s). D2 and D3 showed hyper intensity after reperfusion in each group (Fig 5A).

#### BSFI ratio confirmed SFI ratio results

**Fig 6:**
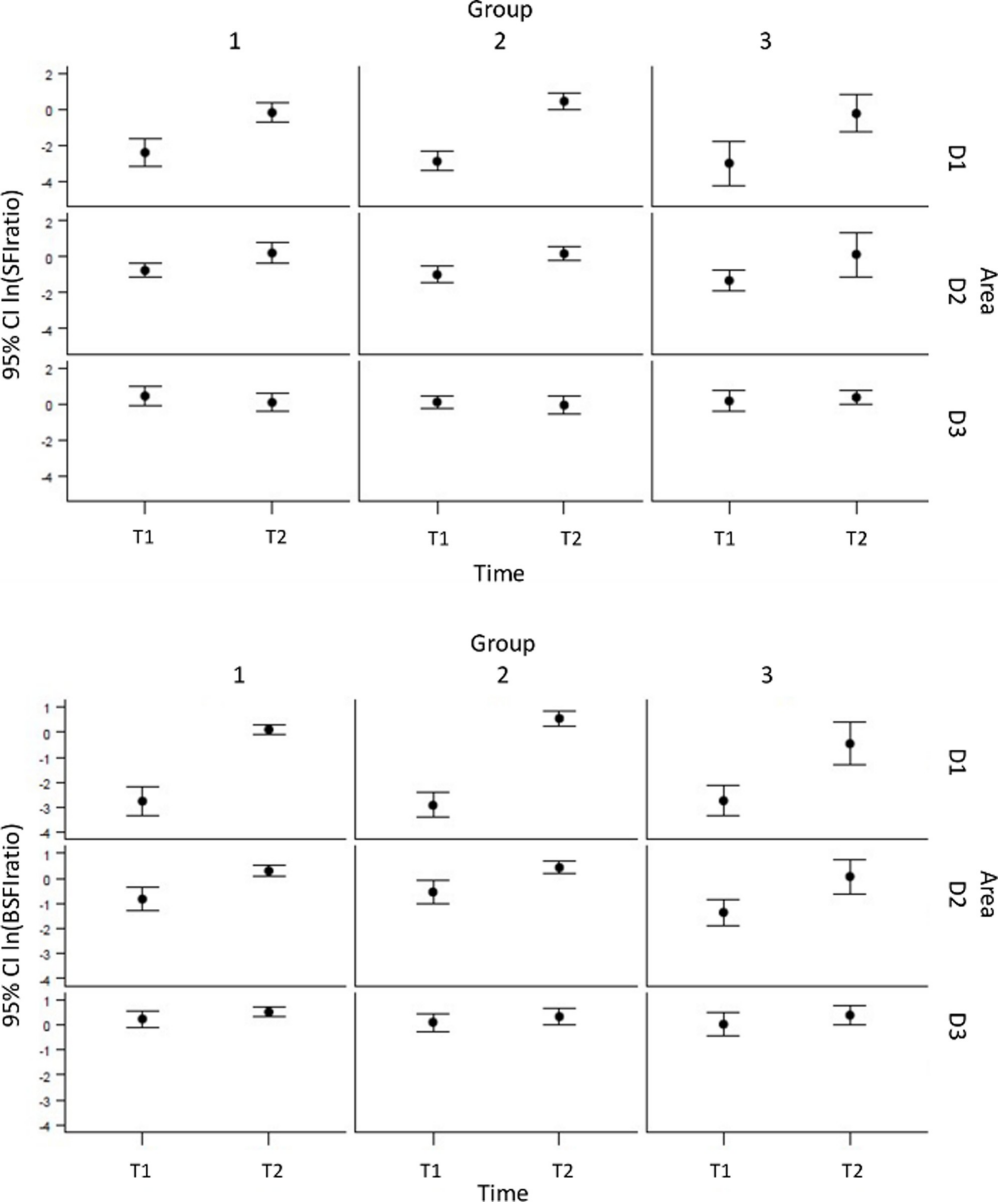
**a.** SFI–Ratio. Under occlusion SFI ratio reflected perfusion deficit in D1 in the different groups. D2 showed impaired perfusion in all groups, while D3 showed hyper intensity. After reperfusion perfusion areal D 1 showed moderate hyper intensity in group I, while group 2 showed more than doubled values. Interestingly Group 3 had similar results to group I with moderate hyper intensity. D2 and D3 showed hyper intensity after reperfusion in each group. P-values for fixed effects were significant for area (pa), time (pt), and time dependent area (pa*t). The graph shows means and 95% confidence intervals of ln-transformed data by area and time. **b.** BSFI–Ratio. Perfusion deficit in D1 is well demonstrated, D2 showed reduced ICG-FI than baseline values, and D 3 revealed hyper intensity, thus, lower than SFI Ratio. After Reperfusion D 1 ICG-FI assimilated again to baseline value in group I, and group III, while group II showed hyper intensity. D2 and D3 revealed hyper intensity after reperfusion in each group. P-values for fixed effects were significant for area (pa), time (pt), and time dependent area (pa*t). The graph shows means and 95% confidence intervals of ln-transformed data by area and time.

*Occlusion (T1)*. Perfusion deficit in D1 is well demonstrated (group I: 0.14±0.19; group II: 0.07±0.05; group III: 0.09±0.11; p<0.05), D2 showed reduced ICG-FI than baseline values (group I:0.55±0.33; group II: 0.70±0.58; group III:0.31±0.21;p<0.05), and D 3 revealed hyper intensity (group I: 1.42±0.60; group II: 1.28±0.57; group III: 1.15±0.53;p<0.05), thus, lower than SFI Ratio. After Reperfusion D 1 ICG-FI assimilated again to baseline value in group I (1.19±0.67; n.s.), and group III (0.98±0.68; n.s.), while group II showed hyper intensity (1.99±1.05; p<0.05). D2 and D3 revealed hyper intensity after reperfusion in each group (Fig 6A and 6B).

## Discussion

Acute mesenteric ischemia is still a life-threatening disease with a high mortality [25]. Evaluation of bowel vitality is the critical point of surgical treatment [26]. Karliczek et al. evaluated the accuracy of surgeons´ judgement in colorectal anastomosis. They found, that the clinical judgement has low sensitivity and specificity for all kind of anastomosis. Still a second look laparotomy is recommended to estimate true constitution of bowel after mesenteric ischemia [25]. Therefore, objective tools to evaluate intestinal perfusion during first operation are necessary to set correctly the resection margins, and to preserve tissue that has the potential to recover.

This study focused on evaluation of ICG-FI in bowel assessment after different periods of ischemia, trying to predict tissue damage and comparing it to histopathological findings.

ICG-FI to evaluate bowel ischemia has been published in small case series earlier [27–30]. The authors summarize that the ICG-FI technology is easy to use and takes little time. Therefore, it can easily be used in an emergency case [30]. Some authors describe a relevant influence on the intraoperative decision making, although all have only made a visual evaluation of ICG-FI [29, 30] with lack of objective quantitative interpretation.

Although ICG-FI is an increasingly popular method and is also widely used for determination of perfusion status in different procedures, so far there have only been a few attempts to carry out a quantification. In an experimental study inducing mesenteric ischemia of 60 minutes in a rat model Toens et al. compared ICG-FI to radioactive microspheres. They evaluated the increase of fluorescence by using the ROI technique. The results of the ischemic parts were compared to control regions. Although their results turned to be inhomogeneous compared to the microsphere technique, ICG-FI was concluded to be a feasible, reliable, and valid technique for mesenteric blood flow assessment [16]. Behrendt et al. described the intestine perfusion after manual manipulation in a rat model using a perfusion index. They compared the region of interest with the SMA [31].

In a comparable study by Matsui a 30 minutes ischemia was induced in a 2–12 cm bowel segment. Quantification was made by analyzing the absolute fluorescence intensity and the contrast-to-background-ratio. In conclusion, a clinical assessment had high sensitivity, but relatively low specificity. Thus, prediction of survival remained unsecure.

Remarkable, quantitative assessment tools like the maximum fluorescence intensity resulted in only a slightly better bowel evaluation, compared to visual evaluation [15].

In another study, Diana et al. compared ICG-FI to metabonomics, lactate levels, and histopathologic findings using a real time assessment in a porcine model. They used the time to peak in ischemic and non-ischemic areas of intestine for quantification after 15 minutes of ischemia. With their results, they clarify the effectiveness of the ICG-FI [32]. In a survival study in pigs, the same group evaluated the anastomotic region during impaired perfusion. In conclusion, an 75% impairment of perfusion severely affects anastomotic healing, compared to a 25% impairment [17].

In contrast to existing animal studies, the present study evaluates ICG-FI after significantly longer ischemic periods, which are closer to everyday routine. ICG-FI confirmed a significant difference of tissue damage comparing 6 and 10 hours of ischemia, in correlation to histological findings. The clinical interpretation of bowel quality after 10 hours is obvious. The evaluation of tissue damage after 6 hours of ischemia is still challenging. Therefore, quantification of ICG-FI using SFI and BSFI is a helpful tool to predict tissue damage, especially if the visual assessment is difficult. As we could show by comparing to microscopic findings, a hyper intensity in Group 2 dues to a cellular damage causing capillary leakage with successive ICG pooling. This ICG pooling may cause a misjudgment of the perfusion status of the bowel. Already damaged tissue can be estimated as optimal perfused by visual evaluation alone. The pooling stays longer due to loss of epithelial integrity. Excellent perfused tissue has a immediate influx of ICG, and a gradual, but obvious outflow.

To reduce the effect of hemodynamic differences, a ratio to baseline values was established. All side effects, that influence hemodynamics, such as cardiac output, blood pressure, vascular resistance, volume load, catecholamine substitution also affect ICG-FI results and may lead to misinterpretation. These side effects and hemodynamics are important for accurate measurements. In clinical routine a stable experimental is not achievable. Thus, a ratio to a baseline value, or correlation to a non-ischemic area during the same measurement is mandatory for correct interpretation. Until now, the influence of hemodynamics is undervalued.

SFI and BSFI are conclusive evaluation parameters using ICG-FI for detection of tissue viability. Further studies in humans are mandatory to determine the predictive value of ICG-FI assessment.

## Conclusion

This experimental study in a porcine model of mesenteric ischemia gives evidence that ICG-FI is a promising tool for bowel viability assessment after different periods of ischemia. Solely macroscopic evaluation is possible, but not sufficient. Additional quantitative assessment is useful to support intraoperative decision making. Nevertheless, hemodynamic status have to be taken into consideration to avoid misinterpretation of ICG-FI. Thus, observational studies in humans are intended to further consolidate the findings.
